# Meteorological Controls on Local and Regional Volcanic Ash Dispersal

**DOI:** 10.1038/s41598-018-24651-1

**Published:** 2018-05-02

**Authors:** Alexandros P. Poulidis, Jeremy C. Phillips, Ian A. Renfrew, Jenni Barclay, Andrew Hogg, Susanna F. Jenkins, Richard Robertson, David M. Pyle

**Affiliations:** 1 Disaster Prevention Research Institute, Kyoto University, Uji, Japan; 2 School of Earth Sciences, University of Bristol, Bristol, UK; 3 School of Environmental Sciences, University of East Anglia, Norwich, UK; 4 School of Mathematics, University of Bristol, Bristol, UK; 5 Earth Observatory of Singapore, Nanyang Technological University, Singapore, Singapore; 6 Seismic Research Unit, University of the West Indies, Jamaica, Trinidad and Tobago; 7 Department of Earth Sciences, University of Oxford, Oxford, UK

## Abstract

Volcanic ash has the capacity to impact human health, livestock, crops and infrastructure, including international air traffic. For recent major eruptions, information on the volcanic ash plume has been combined with relatively coarse-resolution meteorological model output to provide simulations of regional ash dispersal, with reasonable success on the scale of hundreds of kilometres. However, to predict and mitigate these impacts locally, significant improvements in modelling capability are required. Here, we present results from a dynamic meteorological-ash-dispersion model configured with sufficient resolution to represent local topographic and convectively-forced flows. We focus on an archetypal volcanic setting, Soufrière, St Vincent, and use the exceptional historical records of the 1902 and 1979 eruptions to challenge our simulations. We find that the evolution and characteristics of ash deposition on St Vincent and nearby islands can be accurately simulated when the wind shear associated with the trade wind inversion and topographically-forced flows are represented. The wind shear plays a primary role and topographic flows a secondary role on ash distribution on local to regional scales. We propose a new explanation for the downwind ash deposition maxima, commonly observed in volcanic eruptions, as resulting from the detailed forcing of mesoscale meteorology on the ash plume.

## Introduction

One major impact of explosive volcanic eruptions is the dispersion of volcanic ash particles in the atmosphere^1^ and their deposition on the ground^2–4^. The predominant distribution of deposited ash is a monotonic reduction in thickness and grainsize with distance from the source^5–7^. However, departures from this monotonic thinning are ubiquitous. There can be complex variability in deposition close to the volcano, e.g. strong localised variability in thickness and grain size characteristics^8–10^; and farther from the source, where distal deposits show secondary maxima of deposit thickness^11–13^. Some aspects of this variability have been explained variously by ash particle characteristics^14^, particle-scale processes such as aggregation^15^, and mechanisms such as localised gravitational instabilities^16^; however direct evidence for these mechanisms is often absent or inconclusive. Here we show that many of the key features of proximal and distal volcanic ash deposits can be reproduced using a fully-coupled meteorological-ash-dispersal model, with the critical new insight being that these features can arise solely from the combined effects of the erupted ash particle distribution and local meteorology. This is revealed using a fully-coupled model which avoids the temporal interpolation of meteorological variables (ie. wind, pressure and humidity fields), thus improving model fidelity, as highlighted in recent studies^17–20^.

## Historical Eruptions of St Vincent

La Soufrière, St Vincent in the Caribbean has an unusually rich record of historical volcanic activity with six certain and two possible eruptive episodes recorded over the last 300 years^21,22^. Here, we focus on two exceptionally well documented eruptive episodes typical of the range in intensity of La Soufrière’s explosive activity: a short-lived Vulcanian explosion on 25–26 April 1979^23,24^; and a longer-lived eruption on 7 May 1902^25,26^. The 1979 explosive activity consisted of a series of Vulcanian eruptions lasting for two weeks, with ash dispersal predominantly towards the east. Unusually, the 26 April eruptive plume was dispersed to the south, allowing ash deposition on the proximal topography to be sampled in detail^23^. The 1902 eruption commenced with minor phreatomagmatic explosions on the evening of the 6 May and developed via strengthening explosions into a climactic phase from approximately 1300 to 1700 local time on the 7 May. Ash was mainly transported to the east, reaching Barbados after approximately 3 hours. Ashfall over St Vincent and Barbados continued until about 0100 and 0500 (respectively) on the 8 May, and was documented in detail at both proximal and distal locations^22,23,25^. Key observations of timings and the consequent tephra fallout from these eruptions are summarised in Supp. Table 1.

The meteorological model WRF-Chem^27,28^, configured for the dispersion of volcanic ash^29^, has been used to investigate these two eruptions. The model set up consists of three one-way nested domains encompassing St Vincent and neighbouring islands (Supp. Fig. 2). The initial conditions for the wind field and the thermodynamic structure of the atmosphere are those of a typical trade wind situation^30^, including the presence of a strong wind shear (i.e. a reversal of wind direction with height) at a height between 2 and 5 km above sea level (see Supp. Fig. 3). Simulations were carried out with grid sizes down to 0.5 km. Although the grid size is within the ‘turbulence grey zone’^31^ and is thus insufficient to resolve all turbulent motion^32,33^, with use of boundary layer parametrisation it was seen to be sufficient to resolve local orographic flows and larger scale atmospheric convection here (see Supp. Fig. 5) and in similar studies, both in a research setting^20,34,35^ and operationally^36^. An ash plume with a typical umbrella-type distribution of ash with height commonly used to initialise advection-diffusion models^5^ and a prescribed grain size distribution (GSD), is inserted at the time of eruption and is passively advected by the simulated three-dimensional wind field (for a comprehensive description see ref.^20^). Transport, dispersion, dry- and wet-deposition of the ash particles are simulated with reasonable fidelity^20,29^, although plume dynamics and interactions with the atmosphere^16,35^, particle aggregation^15^ and buoyant spreading of the plume^37^ are not included in the model.

A Control simulation is initialised using the ERA-Interim^38^ and ERA20C^39^ state-of-the-art meteorological reanalyses datasets from the time and date of each eruption. Two sensitivity simulations are also presented: one initialised using meteorological input fields that are similar in terms of thermal structure but different in terms of the wind profile (i.e. an Alternative Wind simulation with lower shear); and one where the topography of the islands has been set to zero (i.e. a Flat Topography simulation). In order to produce realistic and balanced atmospheric fields, the days for the Alternative Wind simulations were chosen subjectively from the ERA-Interim dataset as days with the closest vertical profiles of potential temperature, humidity and wind speed; as well as wind direction profiles over 8 km. The simulations are made with two initial GSDs for each eruption: unimodal representing the GSD at the source, and bimodal based on measurements of the deposit and so including some aggregation of the finer ash particle sizes^23^ (see Supp. Table 3). The experimental setting is summarised in Table 1, while further details on the model and its configuration are in the Methods section and summarised in Supp. Table 2.

**Table 1:** Experimental setting description.

Setting	Date and Time	T (min)	H_P_ (km)	M_R_ (kg s^−1^)	GSD	Reanalysis Dataset	Wind Direction	Topography
1902	07/05/1902 1800Z	180	15	3.2 10^7^	A1,A2	ERA-20C	E/W	Normal
1902	28/12/2003 0600Z	180	15	3.2 10^7^	A1	ERA-I	S/W	Normal
1902	07/05/1902 1800Z	180	15	3.2 10^7^	A1	ERA-20C	E/W	Flat
1979	26/04/1979 0358Z	8	14	3 10^6^	B1,B2	ERA-I	E/N	Normal
1979	27/11/2005 0358Z	8	14	3 10^6^	B1	ERA-I	N/N	Normal
1979	26/04/1979 0358Z	8	14	3 10^6^	B1	ERA-I	E/N	Flat

The columns tabulate the following: (1) eruption setting; (2) date and time of the eruption initiation; (3) eruption duration, *T*; (4) plume height *H*_*P*_; (5) mass eruption rate, *M*_*R*_; (6) grain size distribution used, GSD (see Supp. Table 3 for details); (7) reanalysis dataset used; (8) wind direction below/above inversion (at ~5 km above sea level); (9) state of topography in the simulation.

## Results

### Ash Deposition Patterns

**Figure 1:**
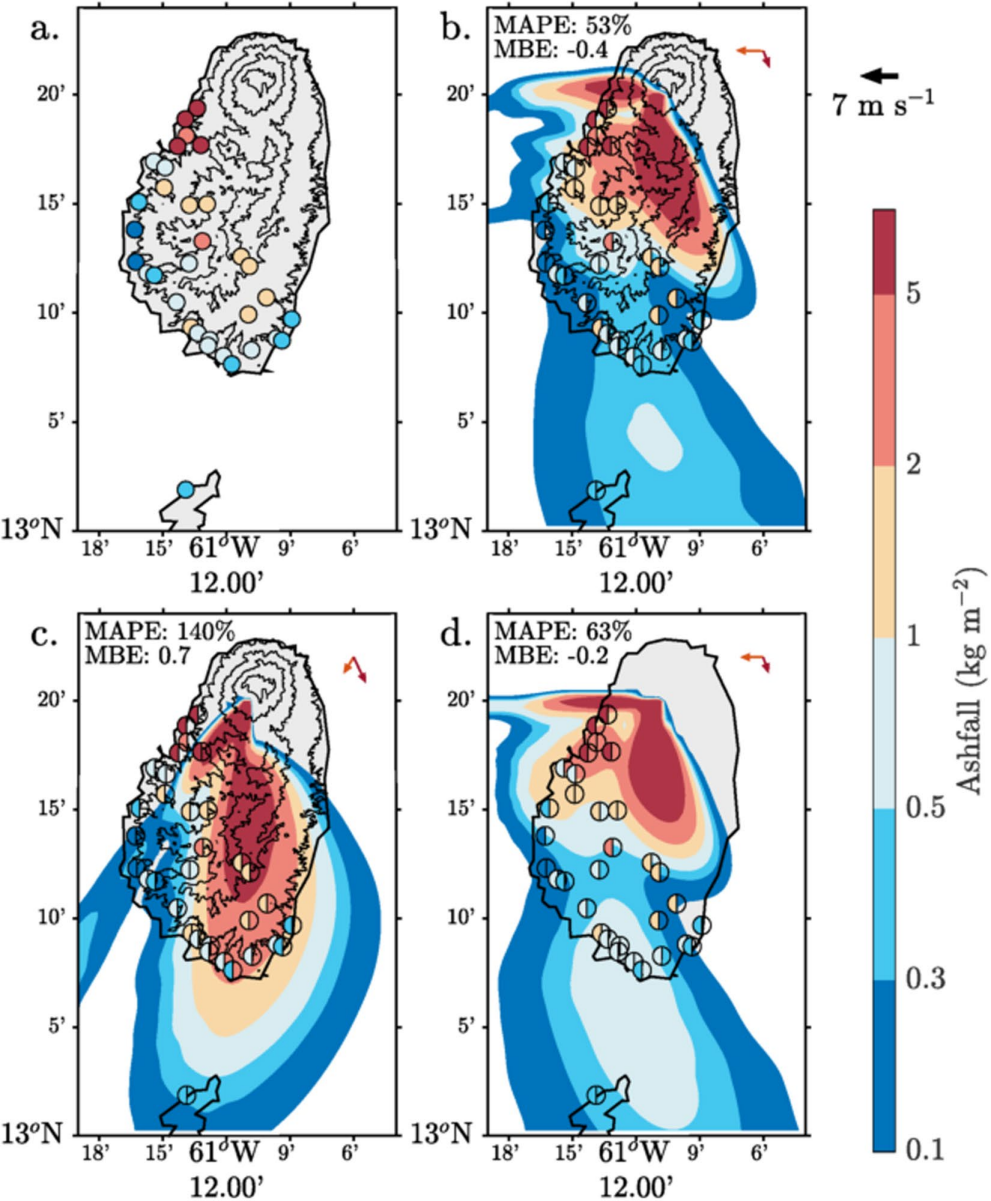
Ash fall distributions following the 26 April 1979 eruption of Soufriere St Vincent. (**a**) Observed, (**b**–**d**) simulated from the meteorological-ash-dispersion model, where the experiments are (**b**) Control; (**c**) Alternative Wind profile (lower shear); and (**d**) Flat Topography. Observations are plotted over the contours using filled circles markers. In Panels b–d observations correspond to the left half of the marker and modelled values to the right. Topographic contours are shown at 100 m and every 300 m after that. Domain-averaged wind vectors at 100 m (orange) and averaged between 6–12 km (dark red) are shown in the top right corner. Plotted using MATLAB Release 2016b, The MathWorks, Inc., Natick, Massachusetts, United States.

The eruption of the 26 April 1979 was marked by atypical meteorological conditions in the upper troposphere: a weak (10 m s^−1^) northerly flow rather than the climatological 20–30 m s^−1^ easterly flow (see Supp. Fig. 3 and ref.^30^). This affected the proximal ashfall distribution with deposition mainly to the south and with a peak on the lee (western) side of St Vincent (Fig. 1a). The Control simulations qualitatively reproduce the proximal ashfall, including the locations of maxima over the western parts of the island and decreased deposition with distance to the south (Fig. 1b). Notable differences with measured ash deposition can be seen in the proximal deposit immediately south of the volcano and on the south coast of St Vincent (simulations are underestimates). Ash dispersal in the Alternative Wind profile simulation occurs along a narrow dispersal axis that, due to the lack of the easterly low-level winds, does not accurately represent ash dispersal over the west side of the island (Fig. 1c). The increased lower level northerly winds in this simulation also result in significant overestimation of the measured deposition to the south of the volcano. Removing the topography causes a subtle redistribution of ash over the island (Fig. 1d). Although this leads to some improvements (especially on the southern coast) it underestimates ashfall inland and overestimates deposition along the western coast. The impact of topographic effects on ash transport deposition has been noted in several studies^11,20,40,41^ and can also be seen in the simulations here: flattening the topography of St Vincent dramatically changes the low-level winds and reduces the amount of turbulent mixing over the island. The Control simulation has strong descent associated with down-slope winds on the leeside of the island, which drives ash towards the ground (see Supp. Fig. 5). This orographic flow descent enhances ashfall by ~10% over St Vincent and by an order of magnitude at some locations (Fig. 1). When compared quantitatively to the observations, the Control simulation has the lowest mean absolute percentage error (MAPE) in ashfall (53.5%) and the lowest corresponding standard deviation (24.5%), while the Alternative Wind profile and Flat Topography simulations degrade the performance of the model so MAPEs are 139.9% and 63.4% respectively. Results here are shown for a unimodal GSD (see Methods). Very similar results and sensitivities are found using the bimodal GSD; the distribution measured including aggregates for this eruption. This suggests that the effects of aggregation are secondary to the high-resolution simulation of lower level winds and orographic flows for the proximal deposition for this eruption. Nonetheless, the source distribution of particles used in a simulation will have a significant influence on proximal deposition patterns, and this likely explains some of the remaining disagreement between modelled and actual deposit measurements for the very proximal situation^5^.

**Figure 2:**
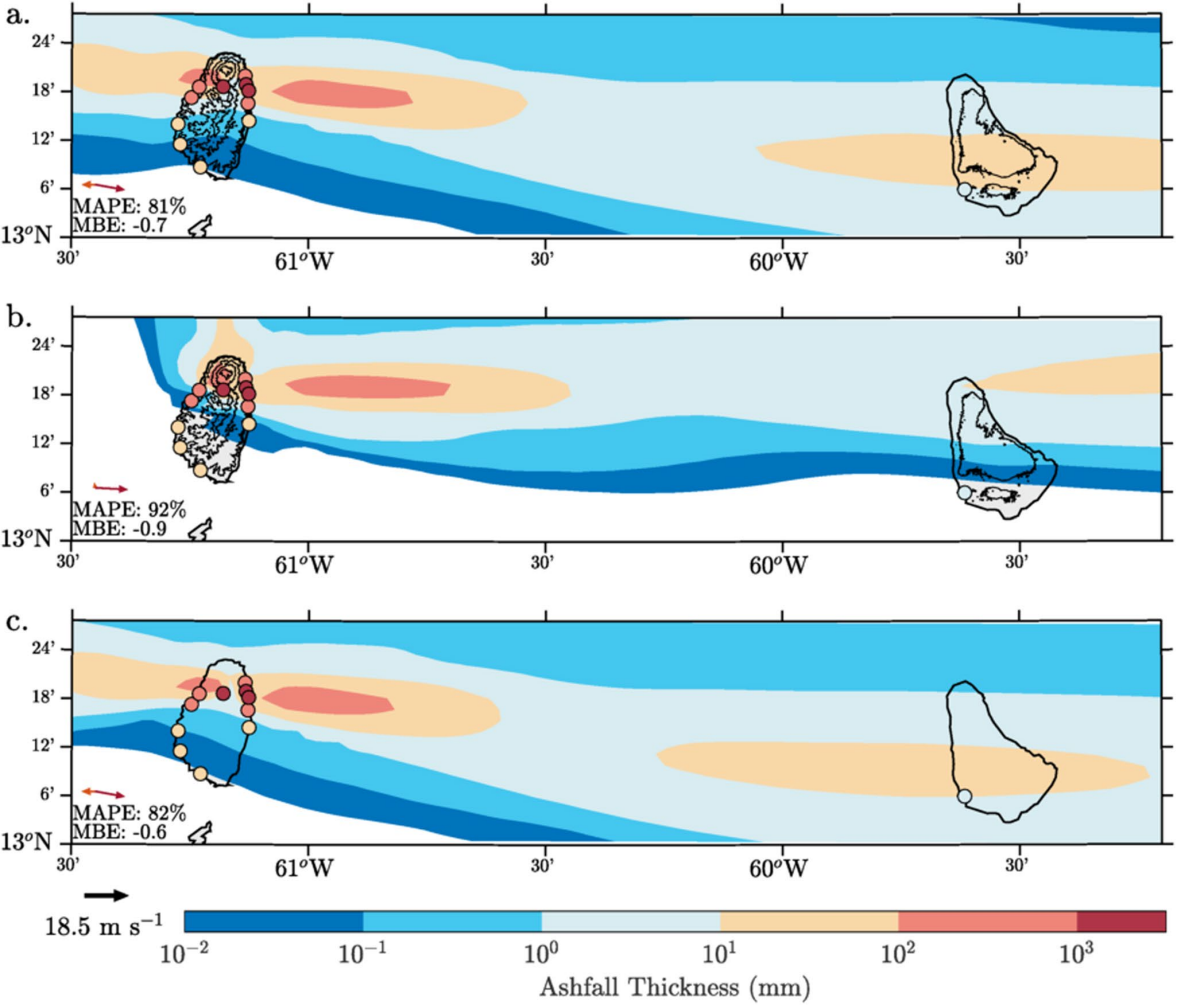
Ash thickness distributions following the 7 May 1902 eruption of Soufriere St Vincent. Simulated from the meteorological-ash-dispersion model where the experiments are (**a**) Control; (**b**) Alternative Wind profile; and (**c**) Flat Topography. Ash thickness observations are plotted over the contours using filled circle markers. Note the ash thickness scale is logarithmic. Topographic contours are shown at 100 m and every 300 m after that. Domain-averaged wind vectors at 100 m (orange) and averaged between 6–12 km (dark red) are shown in the bottom left corner. A deposit density of 1200 kg m^−3^ was assumed to convert model ash fall to ash thickness. Topography contours are shown at 100 m and for every 300 m after that. Plotted using MATLAB Release 2016b, The MathWorks, Inc., Natick, Massachusetts, United States.

The 7 May 1902 eruption (Fig. 2) has a more typical wind profile, low-level easterlies under upper-level westerlies, i.e. a trade wind inversion^30^ (see Supp. Fig. 3). This has produced a contrasting ashfall pattern: the majority of the ashfall is to the east, transported by the upper-level westerly winds, with accumulations of 10–100 mm over St Vincent and Barbados. The Control simulation quantitatively reproduces the ~1000 mm of ash accumulation over the northern part St Vincent and the ~10 mm over Barbados. However, it underestimates the ashfall over the southern part of St Vincent and immediately east of the vent. The most significant underestimation (~2 orders of magnitude) occurs towards the southern coast of St Vincent, revealing the lack of a southwards dispersal component in the model. Historical accounts mention ash reaching the island of Bequia to the south St Vincent but not the island of St Lucia to the north (Supp. Table 1). This evidence suggests buoyant spreading of the plume around its level of neutral buoyancy, with a radius between 30–40 km. Lack of parametrisation for this effect is a common deficiency of ash dispersion models, including WRF-chem. Compared to the 1979 eruption, simulation fidelity is degraded with MAPEs of 81–82% and a mean error bias between −0.7 and −0.6 for the Control and Flat Topography simulations respectively. The Alternative Wind simulation leads to a further decrease in fidelity with a MAPE of 92%. As the detailed dynamics of buoyant spreading remain to be determined^37^, we focus the analysis here on the dynamics that affect ash deposition along the main dispersal axis (ie. east to west). In the Alternative Wind simulation, a small change in the low-level wind direction has a significant impact on the ash distribution, shifting the main ash deposition axis northwards by ~5° (Fig. 2b). This prevents any deposition of ash over the south of both islands, in contrast to what was observed, and drastically reduces the average accumulation over Barbados. Clearly the wind profile has an important role to play in the deposition of ash in both the Control and Alternative Wind simulation. Modest changes in wind shear and wind direction dramatically change the simulated ash deposition. Orographic effects also play a significant secondary role in the simulations, affecting ashfall over both islands by ~10% in 1902 (Fig. 2) and over St Vincent by ~10% in 1979. These results highlight the need for the further parametrisation of buoyant spreading in these models., However, it is also important to note that the underlying dispersal and deposition dynamics discussed in detail in the following sections are, to a degree, unaffected by such changes in the initial ash transport.

Overall, fully-coupled meteorological-ash modelling shows reasonable agreement with deposit measurements for two very different eruptions of St Vincent, in which ash is dispersed in a complex reversing wind field which is strongly influenced by volcanic topography. Some variable agreement between precise proximal deposit measurements and simulation results for the 1979 eruption is expected, given that we have used assumed ash source conditions which are not representative of the actual volcanic plume dynamics.

### Time Evolution of Ash Transport and Deposition

The temporal evolution of the simulated ash deposition over St Vincent for the 1979 Control simulation is similar to that observed^23^. Ashfall begins within a few minutes of model initialisation with 300 g m^−2^ deposited (averaged over the island), ashfall rate peaks at approximately 30 mins and largely ceases after the first hour (see Supp. Fig. 4).

**Figure 3:**
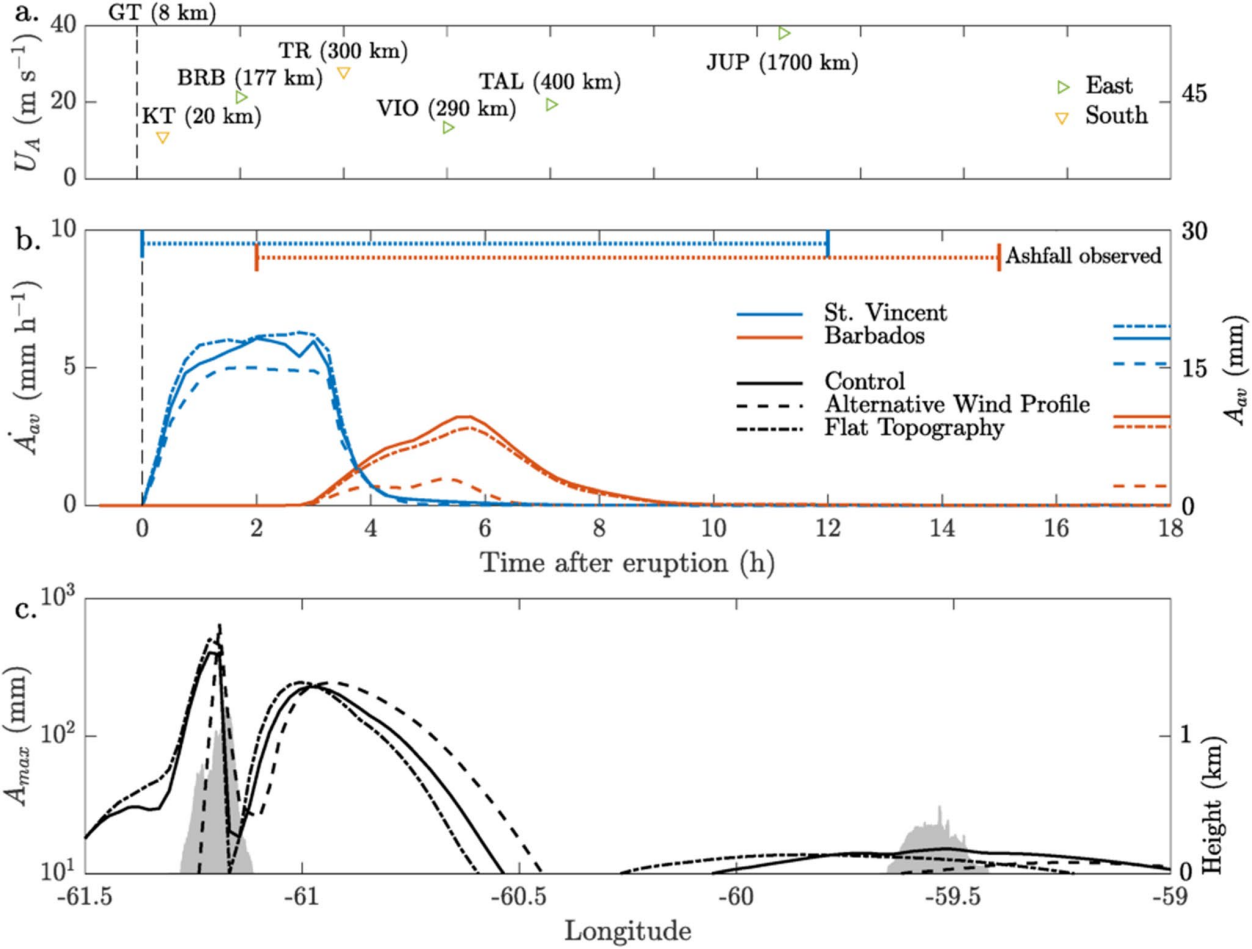
Ash observations and simulations for the 7 May 1902 eruption. (**a**) Estimated ash cloud velocity based on observations of ashfall east and south of the volcano; the numbers in brackets indicate the distance from the volcano. (**b**) Time series of ash thickness change (mm h^−1^) averaged over the two islands for the Control, Alternative Wind and Flat Topography experiments. The final averaged ash thickness (mm) is shown on the right-hand side of the plot. The periods of observed ashfall over the two islands is shown by the dotted lines at the panel top. **(c)** Cross-section of the maximum ash thickness against longitude. Local maxima over St Vincent, east (windward) of St Vincent, and over Barbados are evident. The maximum in topography of the islands is shaded in light grey.

In the 1902 simulations, ashfall over St Vincent starts within the first hour and continues for ~12 hours. The ash cloud reaches Barbados within 3 hours and ashfall peaks at 6 hours, corresponding well with historical records (Fig. 3). Although the timescales for ashfall are similar on both islands, the mass accumulation rate is approximately 3 times larger over St Vincent than over Barbados. Historical accounts of ashfall recorded on islands and on vessels allow us to estimate three ash cloud transport velocities: two eastern velocities of approximately 20 and 40 m s^−1^, and two southern velocities of 10 and 30 m s^−1^ (Fig. 3a). The two eastern ash velocities correspond reasonably well with the wind speeds of the 5–10 and 10–15 km layers in the initialisation profile (Supp. Fig. 3) and here ashfall is simulated accurately, reaching Barbados and the later vessel locations at similar times to those recorded. Note the two southern ash velocities are not captured in the simulations. This lack of reproduction of the 10 m s^−1^ velocity (inferred by the Kingstown observation) almost certainly due to a lack of buoyant plume spreading in the simulations. The 30 m s^−1^ velocity of the southern ash cloud that impacted Trinidad (TR) approximately four hours after eruption onset (Fig. 3a, Supp. Table 1) could be explained by an underestimate of the plume height, or a lack of troposphere/stratosphere exchange, as the only wind component towards the south in the initial wind profile is from an altitude of 19–21 km.

**Figure 4:**
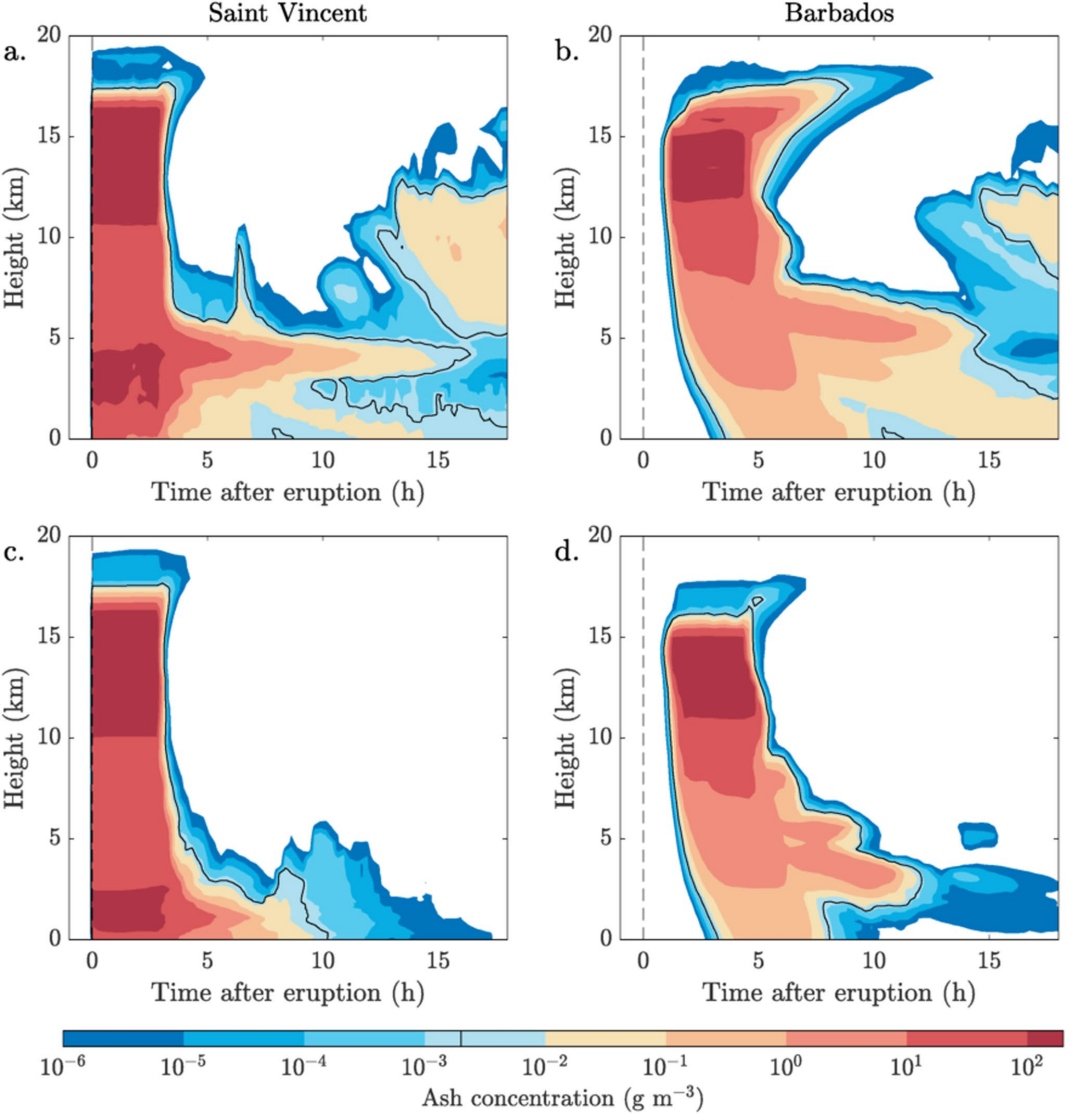
Maximum ash concentration above St Vincent and Barbados over time following the 7 May 1902 eruption. The ash concentration scale is logarithmic and the 2 mg m^−3^ contour (the threat to aviation threshold) is marked in black. The top panels are from the Control simulation and the bottom panels are from the Alternative Wind profile simulation. A lingering hazardous ash cloud is present over St Vincent – and appears over Barbados – throughout the Control simulation, but is not simulated when the wind profile has little vertical wind shear. Plotted using MATLAB Release 2016b, The MathWorks, Inc., Natick, Massachusetts, United States.

Simulations of the spatial and temporal distribution of airborne ash show that the distribution depends strongly on the meteorological conditions. In particular, we find high concentrations of airborne ash particles at the level of the trade wind reversal, about 5 km, where wind speeds are at a minimum. Supp. Fig. 6 shows this ash concentration peak is entirely different in the Alternative Wind simulation, which does not have the wind speed minimum. Over time, the simulated ash cloud evolves very differently in the Control and Alternative Wind simulations (Fig. 4). In the Control simulation, the peak in ash concentration at about 5 km becomes distinct after ~6 hours, consistent with the settling velocity of the median-size particles from their initial positions of highest concentration, leaving a suspended ash cloud over both St Vincent and Barbados for up to ~15 hours after the eruption. The suspended ash cloud is due to a trapping of ash within the low wind-speed layer of the trade wind reversal. It is largely absent from the Alternative Wind simulations (Fig. 4c,d), which do not have the well-defined wind speed minima. This relatively long-lived ash cloud has concentrations above 2 g m^−3^: the threshold for enhanced aviation safety measures and possible disruption to air travel.

### Origins of downwind deposition maxima

All the simulations of the 1902 eruption show multiple ash deposition maxima. Most of the simulated ash dispersal is zonal (Fig. 2), and in a zonal cross-section the simulated ash accumulation has three maxima: over St Vincent, with an enhancement on the leeward slopes and coastal waters; over the sea centred at 61°W; and in the vicinity of Barbados (Fig. 3c). So the meteorological-ash-dispersion model simulates two secondary maxima in ash deposition (in addition to the primary maximum proximal to the volcano). The first downwind deposit maximum (at 61°W) is primarily coarser tephra (2*φ*; 0.25 mm); while the second downwind deposit maximum (at 59.5°W) is primarily medium-sized tephra (3–4*φ*), consistent with observations made on Barbados (Supp. Fig. 7). Here downwind is to the East, so downwind with respect to the upper-level winds.

**Figure 5:**
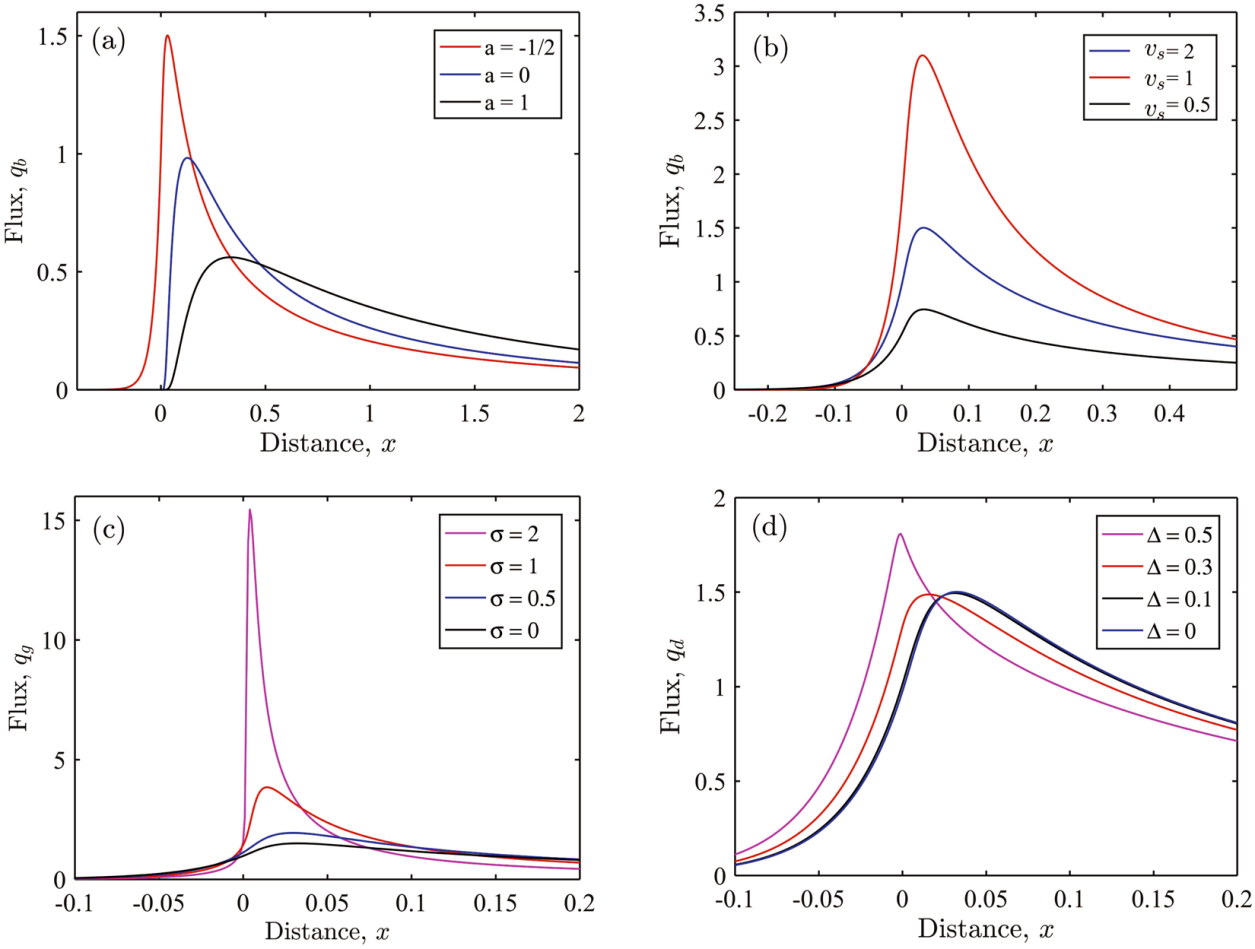
Ash deposition fluxes from an idealised advection-diffusion calculation. Results have been non-dimensionalised and show depositional flux at ground level q_s_ versus distance for different source and wind conditions. Depositional flux for various: (**a**) wind profiles of the form u(z)/U = a + (1 − a)z/H), where U is wind speed at the source height H and a = −1/2 (a trade wind reversal profile); a = 0 (uniform shear); a = 1 (uniform flow); (**b**) settling velocities v_s_; (**c**) grain size distributions, where σ is the standard deviations in *φ* units (the mean *φ* is 5); and (**d**) source thicknesses Δ = Z_s_/H, where Z_s_ is the source layer depth. Default settings use the trade wind reversal profile a = −1/2, a settling velocity v_s_ = V_s_H/κ = 1 (where V_s_ is the particle settling velocity and κ is the turbulent diffusivity), a standard deviation of grain size σ = 2, and a source thickness of Z_s_/H = 0.

To help interpret the underlying deposition mechanisms we employ a set of idealised calculations solving a steady advection-diffusion equation for ash concentration with a prescribed wind profile, settling velocity and source height (details in Methods and Supplementary Information). We find that for conditions relevant for volcanic eruptions a downwind maximum in ash deposition can be obtained from an initial unimodal GSD (Fig. 5). The position of this ash deposition maximum varies with the prescribed wind profile as expected (Fig. 5a) and is not sensitive to the prescribed settling velocity (Fig. 5b). In model runs that fix a trade wind reversal profile, the ash deposition maximum moves upwind as the spread of the GSD increases (Fig. 5c). In this case, since the settling flux is dominated by the larger particle sizes in the distribution, it moves upwind with increasing thickness of the source ash cloud (Fig. 5d), because more of the ash cloud is susceptible to the low-level upwind flow. All of the solutions have a single peak in depositional flux. This suggests that producing multiple downwind maxima in ashfall (as seen in Fig. 3c) would require the superposition of multiple advection-diffusion equation solutions. For example, for a fixed trade wind profile, two maxima would result from two dispersion pathways from two different vertical locations of high particle concentration in the atmosphere, or equivalently from two different ash cloud layer thicknesses (Fig. 5d).

These non-dimensional calculations can be applied to the 1902 St Vincent eruption by noting that the condition σ  = 2 in Fig. 5c has a similar GSD and wind profile to that prescribed in the meteorological-ash-dispersal model. For an advection speed of 40 m s^−1^ and a source height of 10–15 km the calculated idealised deposition flux maximum is about 200 to 300 km downwind – consistent with a maximum near Barbados. For an advection speed of 20 m s^−1^ and a source height of 5 km the calculated deposition flux maximum is about 25 km downwind – consistent with a maximum at around 61°W. These advection speeds are comparable to the wind speeds in the meteorological-ash-dispersal model and the deposition flux maxima are broadly in line with the simulated ash deposition patterns (Fig. 3). The source heights are consistent with medium-size tephra from the plume umbrella (10–15 km) reaching Barbados and a range of tephra sizes from lower in the plume being deposited around 61°W to the east of St Vincent. These idealised calculations demonstrate that when a layer of low wind speed traps ash aloft, this can act as an elevated secondary source of ash particles, and downwind maxima can result directly from the dispersion of this ash without invoking other mechanisms such as particle aggregation^23^ or multiple primary ash sources^14^.

## Discussion

The use of a fully-coupled meteorological-ash-dispersal model to simulate two well-documented historical eruptions of Soufrière, St Vincent provides a novel look into the dynamics of volcanic ash dispersal and deposition both locally and regionally. A configuration of the model with grid sizes down to 0.5 km allows the simulation of flow features and realistic physical processes which affect the dispersal of erupted ash. Misrepresentation of the wind profile, even for the lowest few kilometres from the surface, can cause significant degradation in simulation results (by order 100%). We demonstrate that local and regional-scale ash deposition can be accurately simulated qualitatively, and quantitatively for deposited ash thickness and grain size. Inadequate representation of the orographic or convective-scale meteorology degrades the fidelity of the simulated ashfall by order 10%. We conclude that for accurate simulations and forecasts of proximal and regional volcanic ash dispersal, local meteorological processes need to be represented in detail. However, results here also show that as model resolution increases, the need for the parametrisation of unresolved volcanological processes becomes a priority. In the case of the 1902 eruptions, the lack of buoyant spreading of the plume caused a significant degradation of simulation fidelity, especially for ash deposition outside of the main dispersal axis.

Online ash dispersal calculations eliminate the need for temporal interpolations of the meteorological fields and allow for the study of detailed forcing of the meteorological fields on the volcanic ash particles. As a result, we are able to simulate secondary maxima in ash deposition that resulted from ash cloud trapping in areas of low winds, bounded by high vertical wind shear layers, effectively providing a secondary prolonged source of ash particles, which is subject to different advective and diffusive pathways. These secondary ash deposition maxima are simulated without requiring particle aggregation, particular grain size distributions or separate surface sources. Instead, these maxima arise from the action of meteorological processes on the various ash particles sizes, and this is corroborated by analytical solution of the ash advection-diffusion equation. While these conclusions are drawn from two case studies with relatively complex wind profiles, and differing eruption sizes and spatial scales of ash transport, further studies are needed to generalise the results, and to explicitly include the dynamics of ash aggregation. Overall, our results demonstrate the potential for outputs from research models to offer new insight on the dynamics of ash transport, which can in turn be translated into operational forecast tools in order to better determine arrival times and mass fluxes of airborne and deposited ash.

## Methods

### Numerical Simulations

The chemistry-enabled version of the Weather Research and Forecasting (WRF-Chem) model was used as a meteorological-ash-dispersion model^27–29^. A nested domain approach was employed with an inner domain (∆x = 500 m and 560 (W-E) × 125 (S-N) grid points) focused over St Vincent and Barbados; with intermediate (∆x = 2500 m; 375 × 90) and outer (∆x = 12500 m; 120 × 40) domains covering the broader East Caribbean region (Supp. Fig. 2). All domains had 90 terrain-following vertical levels, with a concentration of levels towards the surface. The topography was derived from the Global 30 Arc-Second Elevation digital elevation model (~900 m resolution) to allow for minimal smoothing^27^, and this was set to zero height for the Flat Topography sensitivity experiments. In Figs 1,2, coastline is shown using the global, self-consistent, hierarchical, high-resolution shoreline (GSHHS) data^42^ and topography is shown based on a Digital Elevation Map (DEM) from the Advanced Spaceborne Thermal Emission and Reflection Radiometer (ASTER) mission^43^.

All the simulations were initialised several hours before the eruption (to allow for a ‘spin up’ of the meteorological fields) and continued for up to 24 h after the eruption. This run length was sufficient for most of the heavy- and medium-size ash (−1–5*φ*; > 2–0.03 mm) to settle and most of the lighter ash (6–8*φ*) to leave the inner domain. Two reanalysis datasets from the European Centre for Medium-range Weather Forecasts (ECMWF) were used for the initialisation and boundary forcing of the meteorological fields: the state-of-the-art ERA-Interim reanalyses (~80 km horizontal resolution)^38^ and the 20^th^ Century ERA20C reanalysis (~125 km horizontal resolution)^39^. This study marks the first time the latter has been used in the context of volcanic ash simulation. Supp. Fig. 3 shows atmospheric profiles from the reanalyses at the times of eruption, as used in the Control (and Flat Topography) experiments. The experimental settings are summarised in Table 1 and the model setup in Supp. Table 2.

Plume dynamics, including buoyant spreading at the plume top, are not included in the model. Instead the plume is inserted instantaneously as an umbrella-shaped distribution over one grid cell and is then passively advected^29^. A 16 km high plume was used for the 1902 eruption^25^. For the 1979 eruption there is some uncertainty on the plume height: reports suggest an 8 km plume^23^, while infra-red images suggest a 14 km plume^44^, which would intrude into the higher wind-shear layer (see Supp. Fig. 3) – this is the value we used. Note the horizontal size of the inserted plume is different for the inner (500 × 500 m) and intermediate (2500 × 2500 m) domain simulations. Comparing the simulations of the larger 1902 eruption, ash thickness from the intermediate simulation is slightly more accurate when compared to the observations, because the larger initial plume size partly compensates for a lack of buoyant spreading. However further model development is required to properly account for buoyant plume spreading^37^.

Two grain size distributions (GSDs) across ten bins were used for each eruption: one unimodal with a peak towards “medium-sized” ash (4–5*φ* for 1902 and 2–3*φ* for 1979; see Supp. Table 3 and Supp. Fig. 7) and one bimodal with an additional peak at −1*φ* for both cases. The unimodal GSD represents the initial erupted ash distribution, while the bimodal GSD can be thought of as a proxy for the aggregation of the finer ash. Ash particle terminal velocity calculations depend on the grain size^45^. The mass fluxes were prescribed as 3.2 × 10^7^ kg s^−1^ for 1902 and 3 × 10^6^ kg s^−1^ for 1979, with the eruptions lasting for 180 and 8 minutes respectively^23,25,46^ (Table 1). To quantify our comparison of simulated and observed ash fall amounts the mean absolute percentage error and mean bias error are shown in Figs 1,2 as they are preferable for variables that range over several orders of magnitude.

A full suite of physical parameterizations was used in the model^27^. Sensitivity tests with different physical parameterization options led to no qualitative differences. Although the WRF-Chem model with online volcanic emissions dispersal can produce realistic simulations of ash distributions^17,20,29^, it does have limitations e.g. particle aggregation is not simulated.

### Advection-diffusion Calculations

We compute the concentration of ash in the atmosphere delivered steadily from a volcanic plume. The suspended ash is advected by the atmospheric winds, settles under gravity and is diffused due to the action of atmospheric turbulence. Here we present calculations from a simple idealised model to draw out the interplay of the dynamical processes and, in particular, to show the ubiquitous feature of a downwind maximum in the depositional flux of ash towards the ground. We analyse the two-dimensional dependence of the concentration field, C(x,z), which satisfies the following dimensionless advection-diffusion equation when the turbulent diffusivity, κ, is constant.

1 $$u(z)\frac{\partial C}{\partial x}-{v}_{s}\frac{\partial C}{\partial z}=\frac{{\partial }^{2}C}{\partial {z}^{2}}+{q}_{s}(x,z)$$

In this expression, vertical distances, z, have been scaled by the height of the steady plume, H; wind velocities by the wind speed, U, at height H; downwind distances by UH^2^/κ; and the concentration field by the dimensionless source strength, q_s_ = Q_s_/(UH). The remaining dimensionless parameter, v_s_, is the settling velocity of the ash (V_s_), scaled by the diffusivity and the height of the plume (v_s_ = V_s_H/κ), and is essentially a Peclet number. In this formulation we have neglected downwind diffusive transport since κ/UH ≪ 1. The concentration field is subject to boundary conditions that require decay far from the ground (C(x, z)→0 as z→∞) and there is only deposition at ground level (∂C/∂z(x, 0) = 0). The key output of the calculation is the dimensionless basal flux of particles, q_b_(x) = v_s_C(x, 0), and we explore its dependence on the wind profile, the distribution of particle sizes and the spatial extent of the source (Fig. 5).

For an eruption on St Vincent, we consider the following order of magnitude estimates for the relevant dimensional parameters: the eruption height, H = 10^4^ m, wind speed at this height, U = 30 m s^−1^, an atmospheric diffusivity κ = 10^2^ m^2^ s^−1^ and a settling velocity V_s_ = 10^−2^ m s^−1^. Together these imply that the dimensionless settling parameter v_s_ = O(1), while the windward length scale is about 3 × 10^4^ km. The wind profile exhibits an approximately constant shear rate, with flow reversal close to the ground. Thus we consider the dimensionless wind speed u(z) = a + (1 − a)z, where a < 1. Notably, a constant wind speed corresponds to a = 1, while the St Vincent data is well fitted by a = −1/2 (Supp. Fig. 3a). Details of the solution are included in Supplementary Information.

### Data availability

The data that support the findings of this study are available from the corresponding author upon reasonable request.

## Electronic supplementary material

Supplementary Information
