# Supplementary material for: Meteorological Controls on Local and Regional Volcanic Ash Dispersal
Source: Sci Rep. 2018 May 2;8:6873. doi: 10.1038/s41598-018-24651-1 (PMC5932072; doi:10.1038/s41598-018-24651-1)
Supplement: Supplementary file 1 — Supplementary Information [file 41598_2018_24651_MOESM1_ESM.pdf]

Supplementary Information for Meteorological Controls on Local and Regional Volcanic Ash Dispersal

Alexandros P. Poulidis[1], Jeremy C. Phillips[2], Ian A. Renfrew[3], Jenni Barclay[3], Andrew Hogg[4], Susanna F. Jenkins[5], Richard Robertson[6], David M. Pyle[7]

[1]Disaster Prevention Research Institute, Kyoto University, Uji, Japan

[2] School of Earth Sciences, University of Bristol, UK

[3]School of Environmental Sciences, University of East Anglia, UK

[4]School of Mathematics, University of Bristol, UK

[5]Earth Observatory of Singapore, Nanyang Technological University, Singapore

[6]Seismic Research Unit, University of the West Indies, Trinidad and Tobago

[7]Department of Earth Sciences, University of Oxford, UK

Contents:

Supplementary Discussion

Supplementary Tables: 3

Supplementary Figures: 7

Supplementary Discussion

Detailed Records for the 1902 Eruption

Time	Observation, (Location), Island or *Vessel*, [Time after Eruption]	Source
**6 May pm**	Initial Explosive eruptions (Multiple places), **St Vincent**	21,25
**7 May** 9am	'Steady showers of dust' (Wallibu), **St Vincent**	25
11am	'a little rain fell containing particles of ash' (Owia) & 'rain began to fall containing particles of ash' (Orange Hills), **St Vincent**	25
12:00-13:00	'small pebbles about a pea-size, commenced to fall', (Georgetown) 'some [stones] were floating on the surface of the water' (Wallibou) 'small stones' (Carib County), **St Vincent**	25
13:00	'the smoke gradually enveloped the whole mountain.' (Chateaubelair) and 'stones began to fall all over the town' (Georgetown), **St Vincent**	47(1.80,1.82)
14:00	Descriptions of advancing pyroclastic density currents over sea, and earlier in Larikai and Wallibou, Lot 14. 'pebbles of a larger size commenced falling' (Richmond Vale) and 'crops were injured but not buried' (Owia) 'darkness then set in fairly rapidly, though by no means instantaneously, and the rain of ash began' (Georgetown), **St Vincent**, **[Eruption (E)]**	25
14:30	'grey pebbles of a pumiceous character..some almost the size of a hen's egg… dry ash followed and this formed the bulk of the shower' (Kingstown), **St Vincent**, **[E+0.5 hours]**	25
14:40	'scoriae of the size of pence and half-pence began to fall thickly in Kingstown' (Kingstown), **St V. [E+0.66h]**	47 (1.80)
16:00	'Lamps had to be lighted about 4 o'clock in the afternoon… the air was so full of falling dust that the appearances resembled a very thick, dark city fog' (Kingstown), **St Vincent [E+2h]**	25
22:00	'a steady rain of volcanic dust has last until the present time, though much lighter now than at first' (Kingstown), **St Vincent [E+8h]**	47 (1.80)
14:15	'cannonading was heard rising to a continuous roar... a rain of fine impalpable ash continued all the afternoon, and, presumably most of the night', **Bequia [E+0.25h]**	25
14:15	'no considerable fall of ashes and no darkness, but only a slight haze in the atmosphere… the amount…as to form only a thin film, the thickness of which was too small to be measured.' **St. Lucia [E+0.25h]**	25
16:00	Ash fall on Barbados begins, **Barbados [E+2h]**	47 (1.83)
17:00	'Steady downpour dust on Barbados', **Barbados [E+3h]**	47 (1.9)
17:15	'at 5.15 the dust was falling fairly fast. The dust shower increased in intensity, and at about 7 p.m was quite heavy the particles falling with a low hissing sound' 13 ounces per square foot [ie. 0.3 kg m^{-2} or 2.5mm] from 17:15 to 05:00, **Barbados [E+3.25h]**	25
19:00	'At first the fall was slight and the substance was gritty and coarse than that which fell afterwards. By 7 o'clock a powdery thick dust was falling thick and fast.'… 'at daybreak was perceived to lie full half an inch thick [12.7 mm] on the surface of the ground' Barbados, **Barbados [E+5h]**	25
	'slight fall of fine dust' Port of Spain, **Trinidad [E+3–5h]**	25,47
20:00	'met the dust', (***Violo*****, 70 miles S.E. Barbados**) **[E+6h]**	25
22:00	'met the dust', (***Talisman*****, 150 miles, SSE Barbados**) **[E+8h]**	25
**8 May** 02:30	'dust fell on the decks', (***Jupiter*** **record, 830 miles ESE Barbados)**	25
05:00	Ash fall on Barbados ceases, **Barbados [E+15h]**	47 (1.83)

Supplementary Table 1 | Historical eruption and ash fall observational records around the 7 May 1902. The columns tabulate: (1) the time; (2) the historic observation, the location and the time after Eruption (E) as set in the simulations; and (3) the source of the historic information. See Supplementary Fig. 1 for locations on St Vincent.

Supplementary Table 1 shows a compiled list of references to the ash cloud and ash deposition from various historical references. Before the main eruption light ashfall was reported at a number of locations over the northern part of St Vincent (see Supp. Fig. 1). After the main eruption started (at 14:00), ashfall was first reported at the central part of the island (Georgetown) and at the southern coast (Kingstown) within 30 minutes. Ashfall was reported over the island of Bequia and Trinidad to the south, but not over St Lucia to the north. The volcanic cloud was mainly dispersed to the east, with reports on Barbados starting at 16:00, and then at various vessels east of Barbados at 20:00, 22:00 and 02:30 on the following day.

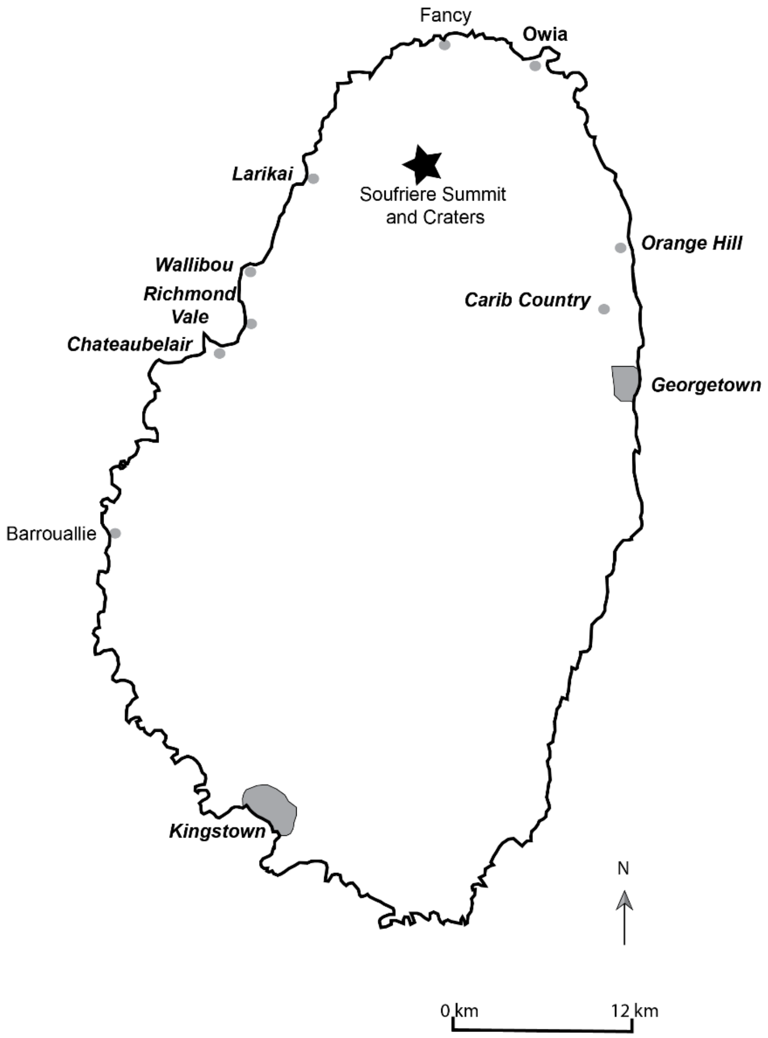

Supplementary Figure 1 | Map of St Vincent, showing the locations of towns and villages impacted by the Soufrière St Vincent eruptions. Locations in bold are mentioned in the text or Supp. Table 1. Redrawn from Brazier et al.[23] using Adobe Illustrator.

WRF-chem Domain Description and Initialisation

The meteorological model WRF-Chem[27,28], configured for the dispersion of volcanic ash[29]. Three one-way nested domain were used (Supp. Fig. 2), utilising a full suite of physical parameterizations (Supp. Table 2).

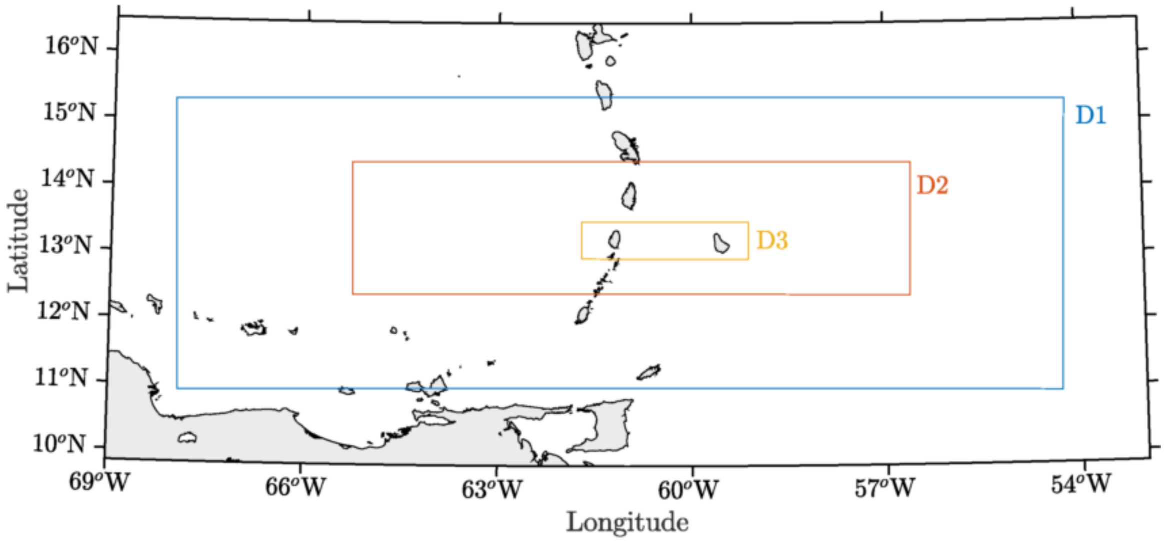

Supplementary Figure 2 | Simulations domains. Coastline is shown using the global, self-consistent, hierarchical, high-resolution shoreline (GSHHS) data[42]. Plotted using MATLAB Release 2016b, The MathWorks, Inc., Natick, Massachusetts, United States.

Domain	*Δt* (s)	Grid number	*Δx* (m)	MP	SW/LW Radiation	LS	SL	PBL	Cu	Ash
D1	75	121x41x91	12500	Lin[48]	Dudhia[49],RRTM[50]	Noah[51]	MM5[52]	MYNN[53]	KF[54]	10-bin, passive[29]
D2	15	376x91x91	2500	Lin	Dudhia,RRTM	Noah	MM5	MYNN	-	10-bin, passive
D3	3	561x126x91	500	Lin	Dudhia,RRTM	Noah	MM5	MYNN	-	10-bin, passive

Supplementary Table 2 | WRF-chem physics and parametrisation options. The columns tabulate: (1) Domain; (2) Time step, *Δt*; (3) Domain size, East-West x South-North x Vertical; (4) Horizontal grid spacing, Δx; (5) Microphysics scheme, MP; (6) Short- and Long-wave radiation scheme, SW/LW Radiation; (7) Surface Layer scheme, SL; (8) Planetary Boundary Layer scheme, PBL; (9) Cumulus parametrisation scheme, Cu; (10) Volcanic ash scheme.

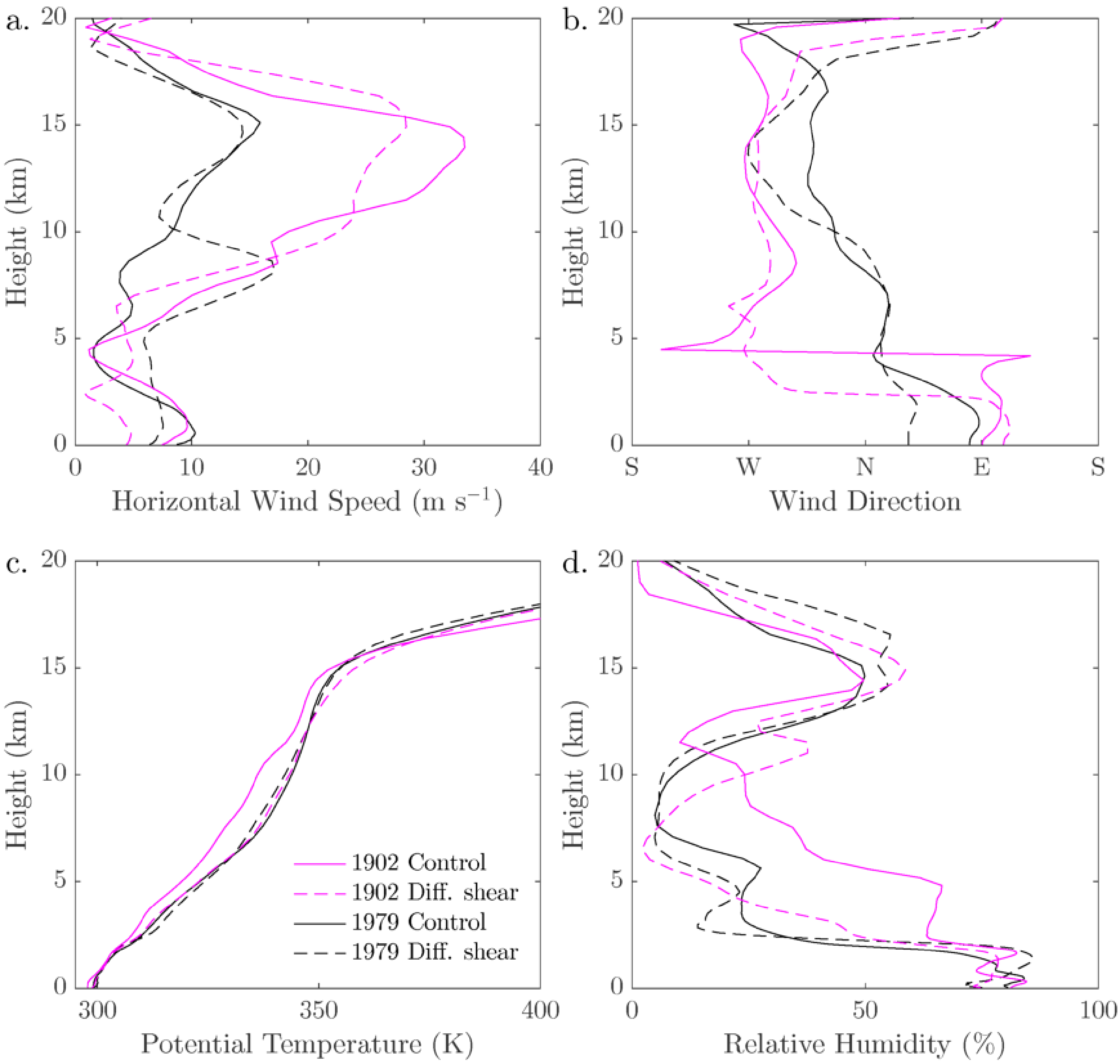

Supplementary Figure 3 | Atmospheric profiles for the 1902 (pink) and 1979 (black) eruptions. The profiles are extracted from the initialisation data (ERA20C for 1902 and ERA-Interim for 1979). The Control profiles are for the time of the eruption, on 7 May 1902 and 26 April 1979, the Alternative Wind profiles were subjectively selected as having similar potential temperature and humidity profiles, but different wind profiles.

Supp. Fig. 3 shows atmospheric profiles over the St Vincent grid cell from the meteorological reanalyses data used to initialise the model. Meteorological reanalyses assimilate observations into a fixed numerical weather prediction system to generate a consistent optimal estimate of the state of the atmosphere at that time. The ERA-Interim and ERA20C reanalyses are available at grid resolutions of ~80 and ~125 km respectively, so the Caribbean islands are all sub-grid scale. The profiles used here are from 00 UTC 26 April 1979 and 18 UTC 7 May 1902 (Control profiles) and 00 UTC 27 November 2005 and 06 UTC 28 December 2003 (Alternative Wind profiles). In 1902 the eruption occurred during typical trade wind conditions[30], with a strong wind shear with height evident; while the Alternative Wind profile has reduced surface easterlies. In 1979 the 26 April eruption (Control profile) occurred during relatively unusual wind conditions at upper levels, only moderate winds and with a northerly component; while the Alternative Wind profile has reduced surface easterlies and a westerly component aloft. Initialising the model with accurate meteorological fields is vital for an accurate simulation of ash fall, as evident from Figs. 1 and 2, where it is clear that relatively small changes in the wind profile lead to dramatically different ash distributions.

Results

1979 Eruption

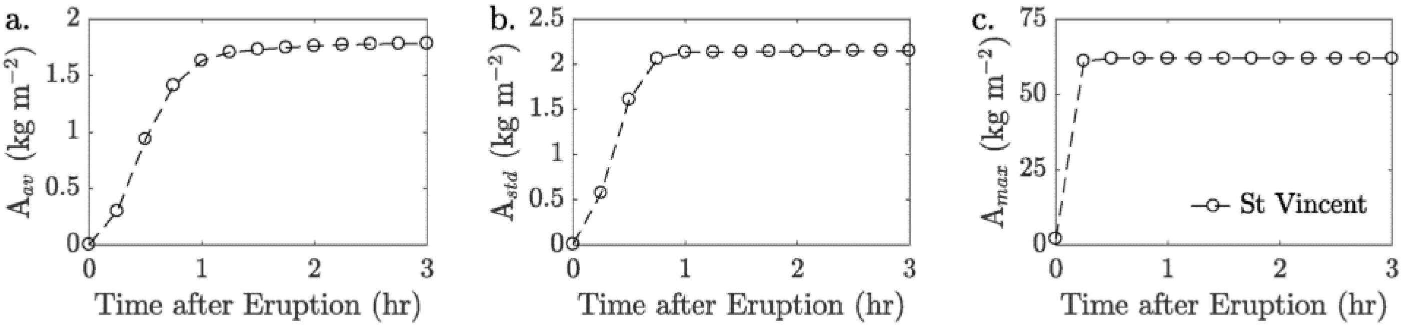

Supplementary Figure 4 | Time evolution of ashfall during the 1979 Control simulation. Results shown for: **(a)** Average ashfall over St Vincent; **(b)** Ashfall standard deviation; **(c)** Maximum ashfall.

The temporal evolution of the simulated ash deposition over St. Vincent for the 1979 Control simulation is similar to that observed[23] (Supp. Fig. 4). Ashfall starts increasing shortly after the eruption, peaks at 30 minutes after the eruption and ceases shortly after the first hour (Supp. Fig. 4a). Standard deviation values are of the same order of the averages, showing that ash covered the island non-uniformly (Supp. Fig. 4b). Despite the gradual increase in the average ashfall, the largest ashfall accumulation was achieved within 15 minutes of the eruption, due to the deposition of the heaviest ash bins (Supp. Fig. 4c).

Appropriately configured numerical weather prediction models are capable of simulating the atmospheric flow around small mountainous islands with a high degree of fidelity[55-57]. As flow impinges on a barrier a number of topographic phenomena are forced depending on the flow speed (U), the atmospheric stability (commonly shown via the Brunt-Väisälä frequency, N) and the mountain topography (ie. mountain height, H). The Froude number (Fr=U/NH) is commonly used to characterise a flow based on the expected orographic effects[59]. Here, for the period after the eruption Fr varies between 0.64-0.84 for the northern peak and 0.98-1.28 for the rest of the island. Over the northern part of the island some flow distortion around the volcano is expected (ie. flow blocking), while over the southern part phenomena such as acceleration on the leeside (downslope wind storms), leeside wakes and internal gravity waves are expected[59]. The simulations resolve the expected phenomena, which are all visible in Supp. Figs. 5a,b and 6a,c, and absent in Supp. Figs. 5c and 6e. The down-slope winds and associated internal gravity waves triggered over St Vincent are responsible for strong descent (order 1 m s^{-1}) over much of the leeside of the island. This descent will drive ash downwards. It is comparable in size to the gravitational settling velocity that is also acting on the tephra, so provides a significant enhancement to local ash fall. Comparing panels (b) and (d) in Fig. 1 demonstrates this enhancement, showing increases ash fall locally by an order of magnitude and by ~10% over the whole island. The impact of the mountain on the flow also generates significant amounts of turbulence in the atmosphere, evident through large turbulent kinetic energy (not shown). This enhanced turbulence will mix the atmosphere, for example, reducing static stability and effecting the three-dimensional wind field. The magnitude (order 1 m s^{-1}) of the orographically-induced descent over St Vincent makes it important for the model to resolve such features and implies a high horizontal resolution is needed. In the Alternative Wind simulations (Supp. Fig. 5d,e), the change in the low-level wind direction enforces a completely different local circulation over the island, with ascent over the northern part and gravity waves propagated across the length of the island.

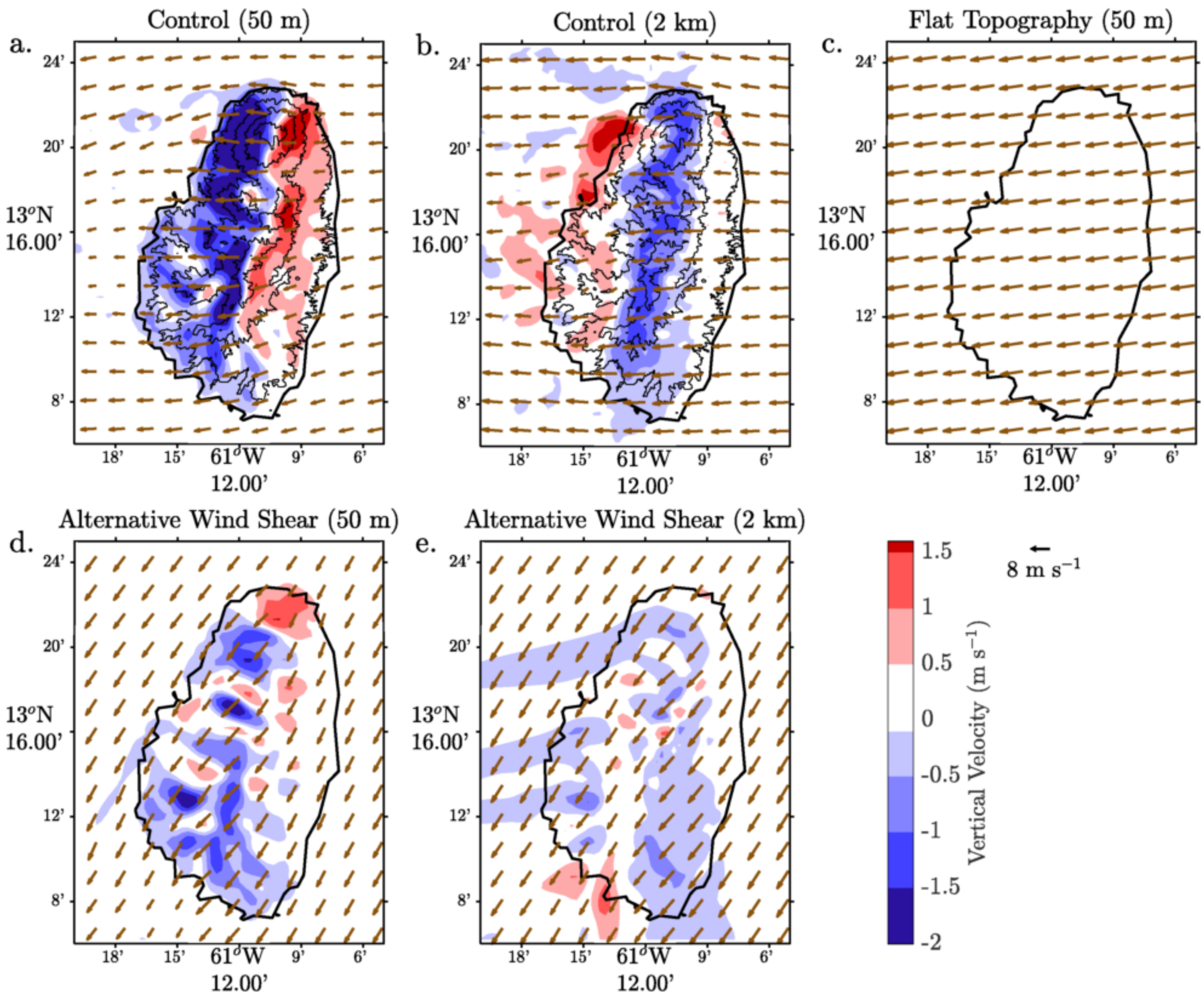

Supplementary Figure 5 | Wind vectors and vertical velocity simulated for the 26 April 1979 eruption. Results are for the: **(a)** Control simulation at 50 m altitude; **(b)** Control simulation at 2 km; **(c)** Flat Topography simulation at 50 m; **(d)** Alternative Wind simulation at 50 m altitude; **(e)** Alternative Wind simulation at 2 km. Horizontal winds are shown as vectors, while vertical velocity is shaded. All quantities are 6-hour averages from the time of the eruption. Topography contours are shown at 100 m and for every 300 m after that. Coastline is shown using the global, self-consistent, hierarchical, high-resolution shoreline (GSHHS) data[42] and topography contours are based on a Digital Elevation Map (DEM) from the Advanced Spaceborne Thermal Emission and Reflection Radiometer (ASTER) mission[43]. Plotted using MATLAB Release 2016b, The MathWorks, Inc., Natick, Massachusetts, United States.

1902 Eruption

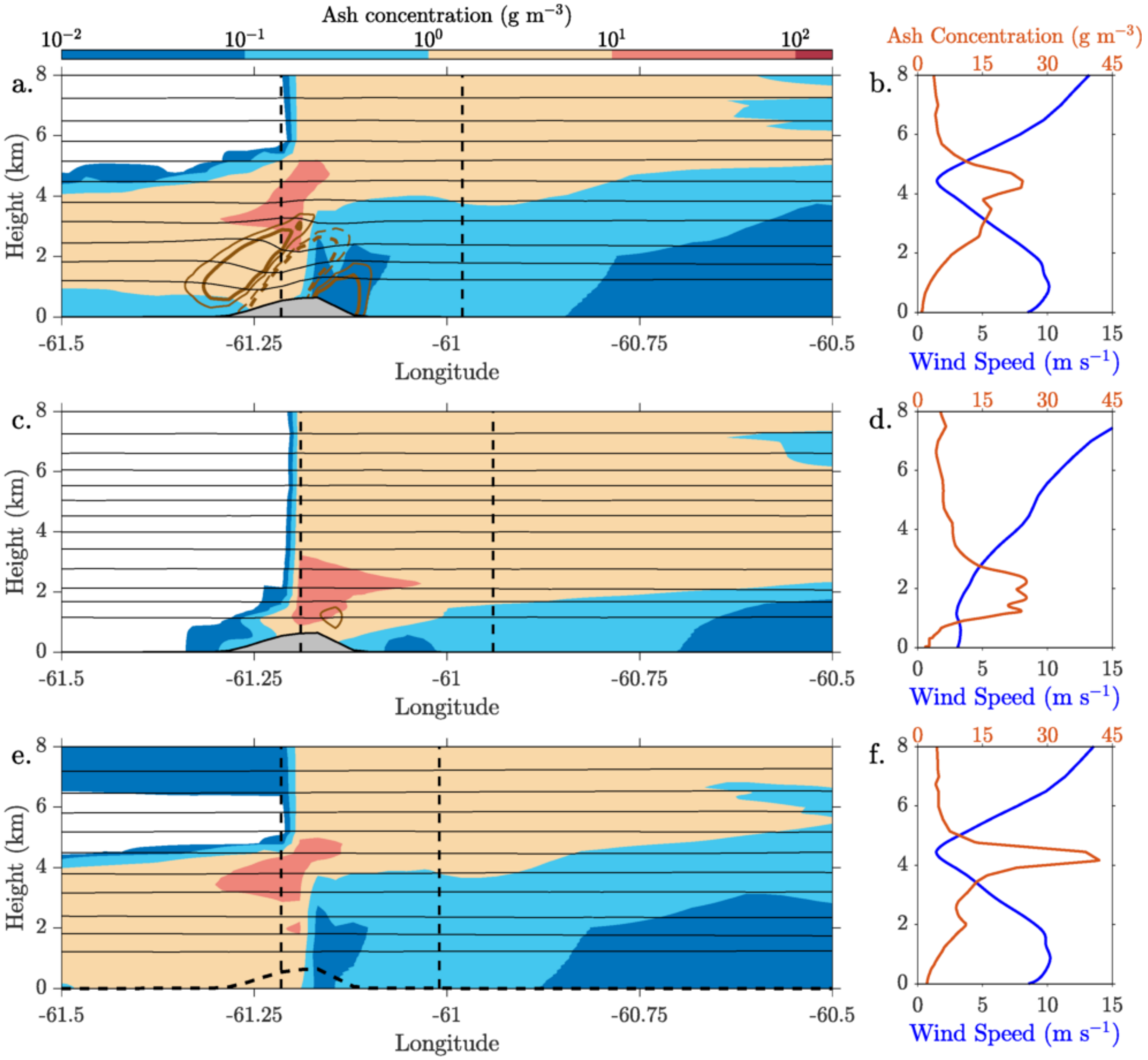

Supplementary Figure 6 | Ash concentration, vertical velocity and wind speed in the vicinity of St Vincent for the 7 May 1902 eruption. The left-hand column shows vertical cross-sections of ash concentration (shaded) and potential temperature (thin black contours) for the **(a)** Control; **(c)** Alternative Wind profile; and **(e)** Flat Topography simulations. Potential temperature contours are shown from 298-320K in increments of 2K. The vertical black dashed lines mark the position of the two ashfall maxima closest to St Vincent for each case. The right-hand panels show averaged: horizontal wind speed (blue) and ash concentration (orange) profiles for the **(b)** Control; **(d)** Alternative Wind; **(f)** Flat Topography simulations. All quantities are 6-hour averages from the time of the eruption.

Supplementary Fig. 6 shows the relationship between local meteorological features and the distribution of volcanic ash. In the Control and Flat Topography simulations the ash cloud spreads to the west at low-levels and to the east higher up. In the Alternative Wind simulation there is little ash to the west as the low-level easterly winds are very weak. The location of the primary and a secondary ashfall maximum coincides with elevated ash concentrations. In the Control simulation some ash is lifted on the leeside, within ascent associated with the orographically-induced gravity wave (indicated by the alternating positive and negative vertical velocities). Gravity waves are not seen in the Alternative Wind or Flat Topography simulations. The gravity waves illustrated here for the 1902 case are qualitatively

similar to those of the Control simulation for the 1979 case (not shown). Indeed, they are ubiquitous for flow impacting mountains of this size and aspect ratio[55-57,59].

There is a peak in ash concentration (for the height range shown) in all the simulations (see the left-hand panels). This peak is coincident with the wind speed minimum associated with the trade wind reversal in the Control and Flat Topography simulations. This low wind speed layer acts to trap ash and consequently provide an elevated secondary source of ash particles. This contributes to the presence of the secondary ash deposition maxima at 61°W and around Barbados (see Figs. 2 and 3). Without accurate representation of the meteorology this elevated secondary source does not exist (e.g. see the Alternative Wind simulation). It is critical for the lingering suspended ash cloud between St Vincent and Barbados, illustrated in Fig. 4, which has concentrations that would be a hazard to aviation. In the Alternative Wind profile simulations these meteorological-ash interactions are altered, there is reduced vertical wind shear, with only a slight wind speed minimum around 1.5 km, leading to a broader peak in ash concentration between 1 and 3 km. There is less ash above this height and so reduced suspended ash transport to the east (see Fig. 4) and a change in the location of the secondary maxima; for example, barely impacting Barbados (Fig. 3) in contrast to the observations.

Grain size distributions

Measurements of the 1979 eruption deposits on St Vincent[23] showed a bimodal GSD for the early part of the eruption, and observations of ash falling as aggregates (accretionary lapilli), of which about 10% were preserved on landing. The aggregate GSD was similar to the fine fraction of the GSD of the later phases of the eruption, and a unimodal total GSD was reconstructed from these measurements[23]. Measurements of 1902 eruption deposits showed some preservation of accretionary lapilli in proximal deposits, and here we use a measured bimodal GSD[58] and a reconstruction of the unimodal total GSD as for the 1979 eruption.

φ	**-1**	**0**	**1**	**2**	**3**	**4**	**5**	**6**	**7**	**8**
A1	0	4.1	4.1	7.7	13.6	20.4	20.6	13.6	7.7	8.2
A2	37.3	3.8	4.8	6	8.7	11.4	11.5	7.6	4.3	4.6
B1	4.3	9.2	14.4	18.5	18.4	16	12.5	5.5	1.2	0
B2	10.8	13	12.5	12.3	17.9	18	10.6	4.5	0.4	0

Supplementary Table 3 | Grain size distributions used in the simulations. The rows tabulate the percentages per grain size ash bin used for the 1902 eruption experiments using a unimodal (A1) and bimodal (A2) distribution and for the 1979 eruption experiments using a unimodal (B1) and bimodal (B2) distribution. Grain size is shown using the Krumbein scale (i.e. $\varphi=-\log_2(d \text{ in mm})$).

Supplementary Fig. 7 shows the prescribed GSD for the 1902 simulations based upon observed GSDs[58]. As both unimodal and bimodal GSDs are plausible, we simulate all cases with both distributions. The results are qualitatively similar and generally remain in the same rank order in terms of a statistical comparison to observations of ashfall. Only medium-sized ash (3φ and 4φ) is simulated to fall over Barbados, which compares well with historical observations (Supp. Fig. 7). In the Alternative Wind profile simulation only 3φ ash falls over Barbados, which compares less favourably with the observations.

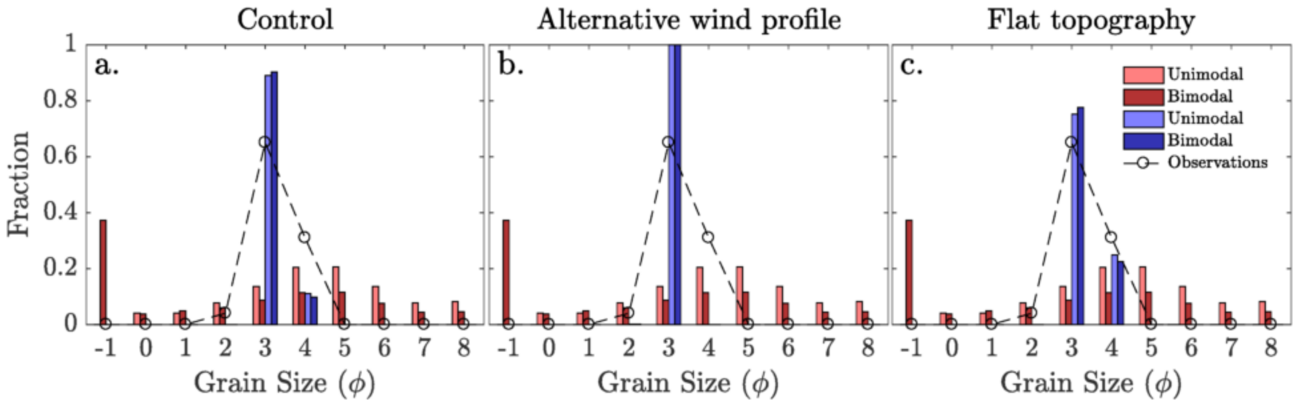

Supplementary Figure 7 | Grain size distributions (GSD) for the 7 May 1902 eruption. The prescribed GSD are shown (light and dark red bars) as well as the simulated GSD over Barbados (light and dark blue bars), for the: **(a)** Control simulation; **(b)** Alternative Wind profile simulation; and **(c)** Flat Topography simulation. Observations made on 7 May[33] on Barbados are shown as circles. Note a grain size of φ=-1 corresponds to >2 mm, φ=0 corresponds to 1 mm, φ=1 to 0.5 mm, etc. Only medium grain sizes (~0.1 mm) are simulated and observed to fall over Barbados: heavier ash falls out before reaching Barbados, while lighter ash remains suspended.

Solution of the advection-diffusion equation

We construct the solutions to Equation (1) analytically using Fourier transforms. When the source is concentrated as (x,z)=(0,h) and $q_s = \delta(x)\delta(z-h)$ (where δ(z) is the Dirac delta function), we find that the settling flux is given by

$$q_b(x;h) = \frac{v_s e^{\frac{hv_s}{2}}}{\pi} \int_{-\infty}^{\infty} \frac{Ai\left(\frac{i\omega(h(1-a)+a)+\frac{v_s^2}{4}}{((1-a)i\omega)^{\frac{2}{3}}}\right)e^{i\omega x}}{v_s Ai\left(\frac{i\omega a+\frac{v_s^2}{4}}{((1-a)i\omega)^{\frac{2}{3}}}\right)-2((1-a)i\omega)^{\frac{2}{3}}Ai'\left(\frac{i\omega a+\frac{v_s^2}{4}}{((1-a)i\omega)^{\frac{2}{3}}}\right)} d\omega \quad (2)$$

where Ai is the Airy function and Ai' its derivative. This integral may be evaluated directly through quadrature and plots of its dependence upon the shear rate parameter, a, and dimensionless settling velocity, v_s, are given in Figs. 5a and 5b. We note that the depositional flux increases with distance from source, reaching a downwind maximum, before progressively decaying in the far-field. This generic distribution occurs for all parameter values. Upwind deposition is possible when a<0, although the bulk of the suspended ash is deposited downwind. The location of the maximum deposition flux moves upwind with increasing settling velocity because the atmospheric turbulence becomes increasingly less able to maintain the particle in suspension.

The effects on the depositional flux of including a grain-size distribution may be readily computed by weighted-averaging the basal flux (2) and by using an expression to determine the settling velocity as a function of grain size[30] To this end, we measure the grain size in terms of the φ-scale such that the diameter in metres is given by $10^{-3}\ 2^{-\phi}$ and we assume that ϕ is normally distributed with mean $\bar{\phi}$ and standard deviation σ. Then the depositional flux is given by

$$q_g(x;h) = \int_{-\infty}^{\infty} q_b(x; v_s, h) \frac{e^{-(\phi-\bar{\phi})^2/(2\sigma^2)}}{2\sigma\sqrt{\pi}} d\phi. \quad (3)$$

We plot this flux as a function of downstream distance for various values of the standard deviation in Fig. 5c. We note that in general the position of the downwind maximum moves upstream as the standard deviation increases. The grain size distribution of the 1902 eruption of St Vincent corresponded to $\bar{\emptyset} = 5$ and σ=2, and these lead to the downwind maximum occurring at x=0.005, corresponding to 200 km downwind, which is in good agreement with simulation results and measurements on Barbados.

Finally we may account for the source no longer being localised by re-writing $q_s = \delta(x)f(z)$ and using (1) which is the Green's function for the concentration field. In this way the depositional flux is given by

$$q_d(x) = \int_0^{\infty} q_b(x; v_s, h) f(h) dh \quad (4)$$

To illustrate these effects, we spread the source uniformly over a vertical layer 2Δ such that $f = (H(1-\Delta) - H(1+\Delta))/(2\Delta)$, where H(z) is the Heaviside step function and plot q_d in Fig. 5d. We note that broadening the source moves the maximum upwind, and for the largest source depths may even move the maximum into x<0 as the settling is strongly influenced by the region of flow reversal close to the ground.

Additional References

47. Blue Book, Correspondence relating to the volcanic eruptions in St Vincent and Martinique in May 1902, with map and appendix. *Parliamentary Paper by Command*, **Cd. 1201**, pp 1-99, HMSO, (Darling and Son, London, 1902).
48. Lin, Y.–L., Farley, R. D. & Orville, H. D. Bulk Parameterization of the Snow Field in a Cloud Model. *J. Climate Appl. Met.*, **22**, 1065–1092 (1983).
49. Dudhia, J. Numerical study of convection observed during the Winter Monsoon Experiment using a mesoscale two-dimensional model, *J. Atmos. Sci.*, **46**, 3077-3107, doi:10.1175/1520-0469(1989)046<3077:NSOCOD>2.0.CO;2 (1989).
50. Mlawer, E. J., Taubman, S. J., Brown, P. D., Iacono, M. J. & Clough, S. A. Radiative transfer for inhomogeneous atmospheres: RRTM, a validated correlated-k model for the longwave, *J. Geophys. Res.*, **102**, 16663-16682, doi:10.1029/97JD00237 (1997).
51. Tewari, M. et al. Implementation and verification of the unified NOAH land surface model in the WRF model, 20th conference on weather analysis and forecasting/16th conference on numerical weather prediction, 11-15 (2004).
52. Monin, A. S. & Obukhov, A. Basic laws of turbulent mixing in the surface layer of the atmosphere, *Contrib. Geophys. Inst. Acad. Sci. USSR*, **151**, 163-187 (1954).
53. Nakanishi, M. & Niino, H. An improved Mellor-Yamada level 3 model: its numerical stability and application to a regional prediction of advecting fog, *Boun. Layer Meteorol.*, **119**, 397-407, doi:10.1007/s10546-005-9030-8 (2006).
54. Kain, J. S. The Kain-Fritsch convective parameterization: An update, *J. Appl. Meteorol.*, **43**, 170-181, doi:10.1175/1520-0450(2004)043<0170:TKCPAU>2.0.CO;2 (2004).
55. Minder, J. R., Smith, R. B., & Nugent, A. D. The dynamics of ascent-forced orographic convection in the tropics: Results from Dominica. *J. Atmos. Sci.*, ***70*,** 4067-4088 (2013).
56. Nugent, A. D., Smith, R. B., & Minder, J. R. Wind speed control of tropical orographic convection. *J. Atmos. Sci.*, ***71***, 2695-2712 (2014).
57. Cécé, R., Bernard, D., d'Alexis, C., & Dorville, J. F. Numerical simulations of island-induced circulations and windward katabatic flow over the Guadeloupe archipelago. *Mon. Weather Rev.*, **142**, 850-867 (2014).
58. Sugden, P. The characteristics and mechanisms of the 1902-3 eruptions of La Soufrière, St. Vincent. M. Sci. Thesis, (University of Oxford, 2015).
59. Smith, R. B. Linear theory of stratified hydrostatic flow past an isolated mountain, *Tellus*, **32**, 348‑364, doi:10.1111/j.2153-3490.1980.tb00962.x (1980).
